# Protein Tyrosine Phosphatase Receptor Type R (PTPRR) Reduces AChR Clustering by Dephosphorylating MuSK

**DOI:** 10.1155/2022/5160624

**Published:** 2022-09-05

**Authors:** Yanxun Chen, Maohao Guan, Fengqiang Yu, Zhongshan Yang, Weiqiang Yi, Xuan Huang, Ruiqin Qiu, Fancai Lai

**Affiliations:** ^1^Department of Thoracic Surgery, Quangang General Hospital, The First Affiliated Hospital of Fujian Medical University, Quanzhou, Fujian Province, China; ^2^Department of Thoracic Surgery, The First Affiliated Hospital of Fujian Medical University, Fuzhou, Fujian Province, China

## Abstract

Neuromuscular junction (NMJ) formation and maintenance depend on the proper localization and concentration of various molecules at synaptic contact sites. Acetylcholine receptor (AChR) clustering on the postsynaptic membrane is a cardinal event in NMJ formation. Muscle-specific tyrosine kinase (MuSK), which functions depending on its phosphorylation, plays an essential role in AChR clustering. In the present study, we used plasmid-based biochemical screening and determined that protein tyrosine phosphatase receptor type R (PTPRR) is responsible for dephosphorylating MuSK on tyrosine residue 754. Furthermore, we showed that PTPRR significantly reduced MuSK-dependent AChR clustering in C2C12 myotubes. Collectively, these data illustrate a negative regulation function of PTPRR in AChR clustering.

## 1. Introduction

The neuromuscular junction (NMJ) is a chemical synapse between motoneurons and muscle fibers and consists of neuronal presynaptic membranes, a synaptic cleft and muscle postsynaptic membranes [1, 2]. The fast and accurate neuromuscular transmissions between presynaptic and postsynaptic membranes rely on highly concentrated acetylcholine receptors (AChRs) at the postsynaptic membranes [3, 4]. In vertebrates, high-density clusters of AChRs are orchestrated by various effector molecules and an intricate network of signaling pathways.

Muscle-specific tyrosine kinase (MuSK), a transmembrane and tyrosine phosphorylated protein, plays a vital role in the clustering of AChRs and is indispensable in both the formation and maintenance of NMJs [5]. MuSK is activated by motoneuron-released agrin, which is prone to aggregation at synaptic basal lamina and binds with low-density lipoprotein receptor-related protein 4 (LRP4) to form the tetrameric agrin-LRP4 complex at postsynaptic membranes [6]. This supercomplex induces the dimerization and autophosphorylation of MuSK. Phosphorylated MuSK recruits intracellular downstream of kinase 7 (Dok-7), which stabilizes and phosphorylates MuSK [7–9]. Tyrosine phosphorylation of MuSK ultimately leads to AChR clustering at postsynaptic membranes.

MuSK exerts physiological functions depending on its phosphorylation. Impairments of MuSK phosphorylation have been associated with several disorders, such as myasthenia gravis (MG) and congenital myasthenia (CMS) [10, 11]. The process of a kinase phosphorylation and dephosphorylation is coordinated in the regulation of signaling responses [12]. As previously noted, the auto- and transphosphorylation of MuSK depends on its dimerization activated by LRP4 and Dok-7. However, which phosphatases dephosphorylate MuSK and mediate the formation of AChR clustering is still unclear.

In the present study, we determined via a plasmid-based biochemical screening that protein tyrosine phosphatase receptor type R (PTPRR) dephosphorylates MuSK on tyrosine residue 754. Furthermore, we showed that PTPRR significantly reduced MuSK-dependent AChR clustering in C2C12 myotubes. Collectively, these data illustrate a negative regulatory function of PTPRR in AChR clustering.

## 2. Results

### 2.1. C2C12 Myotubes Represent an Excellent Experimental Model System for Studying Agrin-Induced AChR Clustering

Prior research has shown that C2C12 myotubes provide an excellent experimental model to examine whether certain synaptic proteins are associated with AChR clustering in muscles. We examined the distribution of AChRs in C2C12 myotubes exposed to increasing concentrations of agrin for 24 h. The agrin concentration gradient (0, 0.3, 0.6, 2.5, 5, 10, and 20ng/ml) was set to determine the optimal concentration. AChRs were visualized and measured via labeling with Alexa Fluor 555 *α*-bungarotoxin (*α*-BTX) for 1 h.

**Figure 1:**
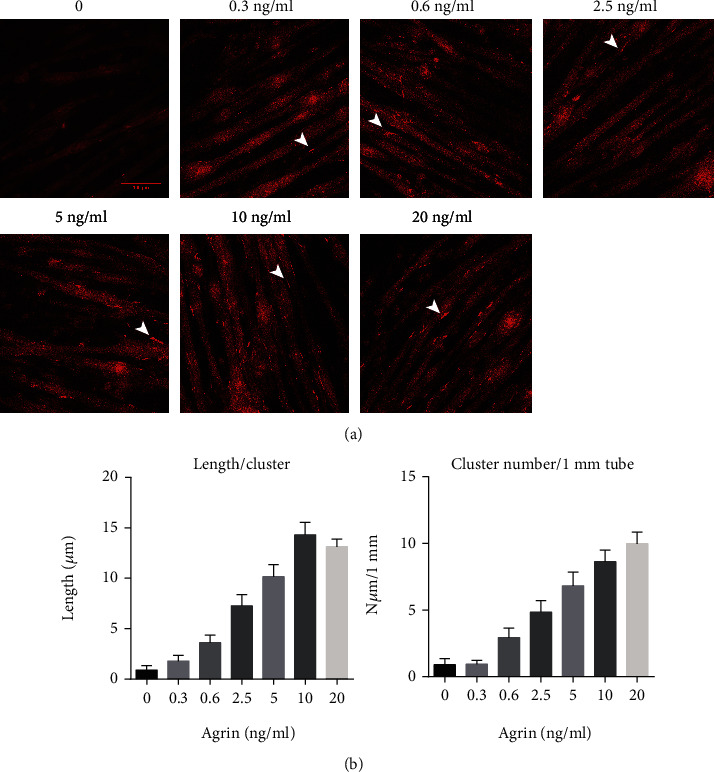
C2C12 myotubes provide an excellent experimental model system for studying agrin-induced acetylcholine receptor (AChR) clustering. (a) Wild-type C2C12 myotubes were incubated with increasing concentrations of agrin (0 to 20 ng/ml) for 24 h, and AChRs were labeled with Alexa Fluor 555 *α*-bungarotoxin. AChR clustering increased with increasing agrin concentrations (0 to 10 ng/ml). C2C12 myotubes treated with 10 ng/ml and 20 ng/ml agrin did not demonstrate a significant difference. White arrowheads indicate AChR clusters. (b) Quantification of AChR clusters ≥5 *μ*m in length from Figure 1. The average length of each AChR cluster and the average number of AChR clusters within a 1 mm tube were quantified. The data were obtained from at least three experiments. Data were shown as mean ± S.D.

As indicated in Figure 1, C2C12 myotubes expressed infrequent AChR clusters (≥5 *μ*m in length) in the absence of agrin. The addition of 0.3 ng/ml to 10 ng/ml agrin induced AChR clustering in a dose-dependent manner. However, C2C12 myotubes treated with 10 ng/ml and 20 ng/ml agrin did not demonstrate a significant difference. Treatment with 20 ng/ml agrin for 24 h increased the AChR cluster length almost 14.7-fold, and the number of AChR clusters (within a 1 mm tube) increased almost 11-fold compared with untreated myotubes (Figures 1(a) and 1(b)). These results indicated that we successfully constructed a C2C12 myotube experimental model system for studying agrin-induced AChR clustering. Furthermore, the optimal agrin concentration was defined as 10 ng/ml, and this concentration was used in the following experiments.

### 2.2. MuSK Is Essential in the Mediation of AChR Clustering

**Figure 2:**
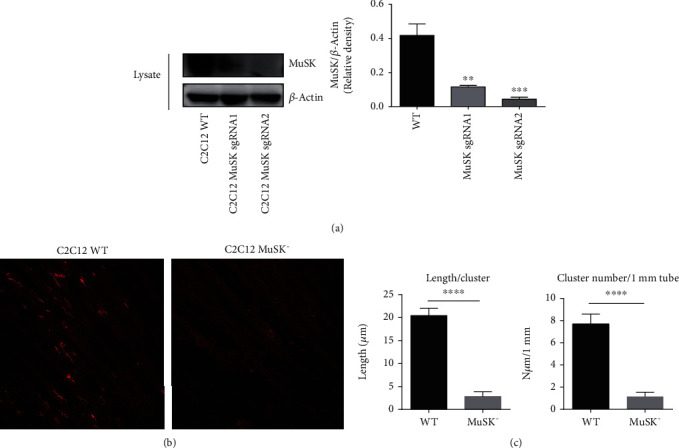
MuSK is an essential molecule that mediates AChR clustering. (a) The efficiency of *MuSK* knockout (*MuSK^−^*) was validated by western blot. MuSK^−^ single clone C2C12 generated by CRISPR sgRNA2 was chosen for the following experiments. (b) Almost no clusters of AChRs were formed in *MuSK*^−^ C2C12 myotubes compared with wild-type myotubes. White arrowheads indicate AChR clusters. (c) Quantification of AChR clusters ≥5 *μ*m in length from (b). The data were obtained from at least three experiments. Data were shown as mean ± S.D. ^∗∗^*P* < 0.01, ^∗∗∗^*P* < 0.001, and ^∗∗∗∗^*P* < 0.0001.

To verify the role of MuSK in agrin-induced AChR clustering, *MuSK* knockout (*MuSK*^−^) single clone C2C12 was constructed using the CRISPR/Cas9 system. The efficiency of *MuSK^−^* was validated by western blot (Figure 2(a)), which showed notable decreased expression in *MuSK*^−^ compared with that in wild-type C2C12. *MuSK*^−^ single clone C2C12 generated by CRISPR sgRNA2 was chosen for this study. After treatment with 10 ng/ml agrin for 24 h, *MuSK*^−^ C2C12 completely abolished the agrin-induced AChR clusters compared with wild-type C2C12 myotubes (Figures 2(b) and 2(c)). These findings suggested that MuSK is essential in the mediation of AChR clustering.

### 2.3. PTPRR Was Identified as the Tyrosine Phosphatases Responsible for Dephosphorylation of MuSK

MuSK serves as a tyrosine phosphorylated protein and is inactivated by phosphatases. Therefore, we speculated that there might be several phosphatases responsible for MuSK dephosphorylation and that participate in the regulation of agrin-induced AChR clustering at postsynaptic membranes.

**Figure 3:**
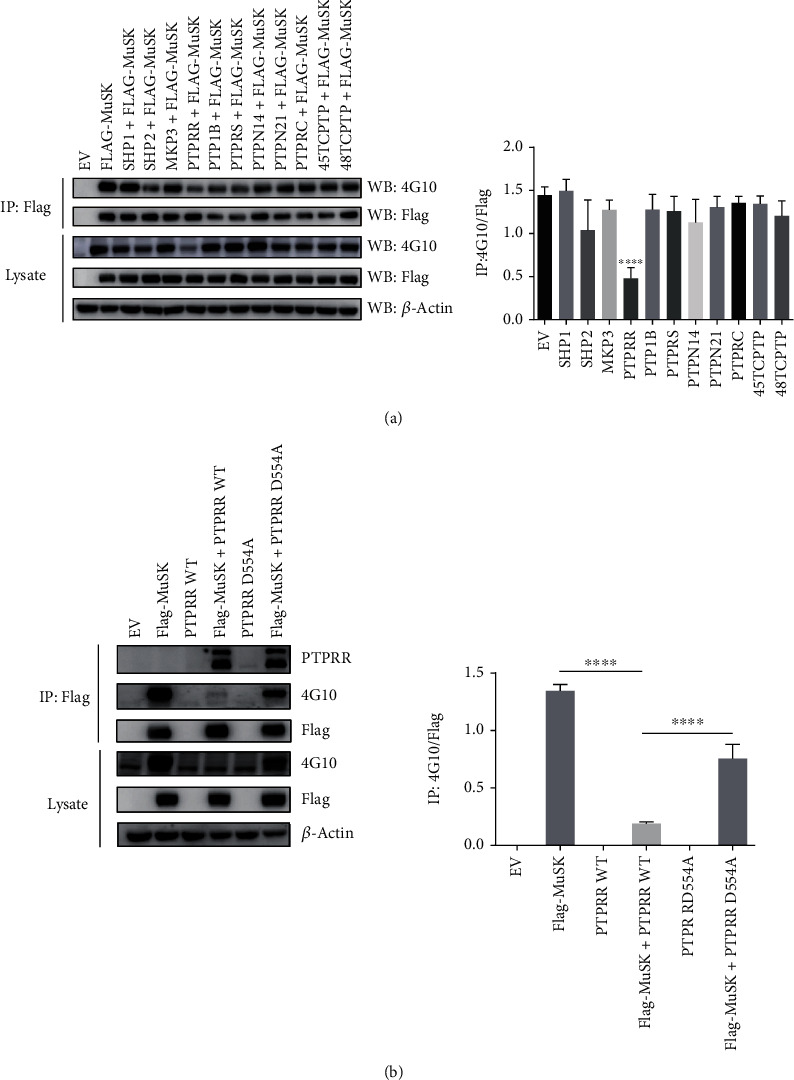
PTPRR was identified as the tyrosine phosphatases responsible for dephosphorylating MuSK via a protein tyrosine phosphatases screening. (a) HEK293T cells were cotransfected with flag-MuSK and 10 various tyrosine phosphatases as indicated. Tyrosine phosphorylation of MuSK and actin were probed. (b) HEK293T cells were transiently transfected with MuSK, PTPRR-WT, or PTPRR-DA mutant. MuSK was isolated by immunoprecipitation with anti-Flag antibody, probed with 4G10 to reveal pY-MuSK, and probed with PTPRR to reveal the interaction between MuSK and PTPRR. Lysates were probed with antibodies against MuSK, PTPRR, and *β*-actin. The data were obtained from at least three experiments. Data were shown as mean ± S.D. ^∗∗∗∗^*P* < 0.0001.

To verify the hypothesis, we performed a protein tyrosine phosphatases screening upon coexpression of 10 classical tyrosine phosphatases with Flag-MuSK in HEK293T cells. An empty vector plasmid (pcDNA3.1) was used as a control. After cotransfection with tyrosine phosphatase for 24 h, the phosphorylation of MuSK cotransfected with PTPRR was significantly decreased compared with that of the empty vector. In contrast, MuSK cotransfected with other phosphatases had relatively high levels of phosphorylation compared with that of the empty vector (Figure 3(a)). The various tyrosine phosphatases were probed and showed in Supplementary Figure S1.

In addition, we generated an Asp to Ala mutation for C-terminal residue 554 of PTPRR. The PTPRR-D554A mutant was a substrate-trapping mutant resulting in PTPRR lacking phosphatase activity. Wild-type PTPRR (PTPRR-WT) and PTPRR-D554A were transiently transfected with MuSK into HEK293T cells. MuSK was purified from cell lysates by immunoprecipitation with Flag-Beads, probed with 4G10 to reveal pY-MuSK, and probed with PTPRR to reveal the interaction between MuSK and PTPRR. Ectopic expression of PTPRR-WT, but not PTPRR-D554A, decreased the phosphorylation level of MuSK (Figure 3(b)). These results suggest that PTPRR may be the tyrosine phosphatase responsible for MuSK dephosphorylation.

### 2.4. PTPRR-Dephosphorylated MuSK-Tyr754

**Figure 4:**
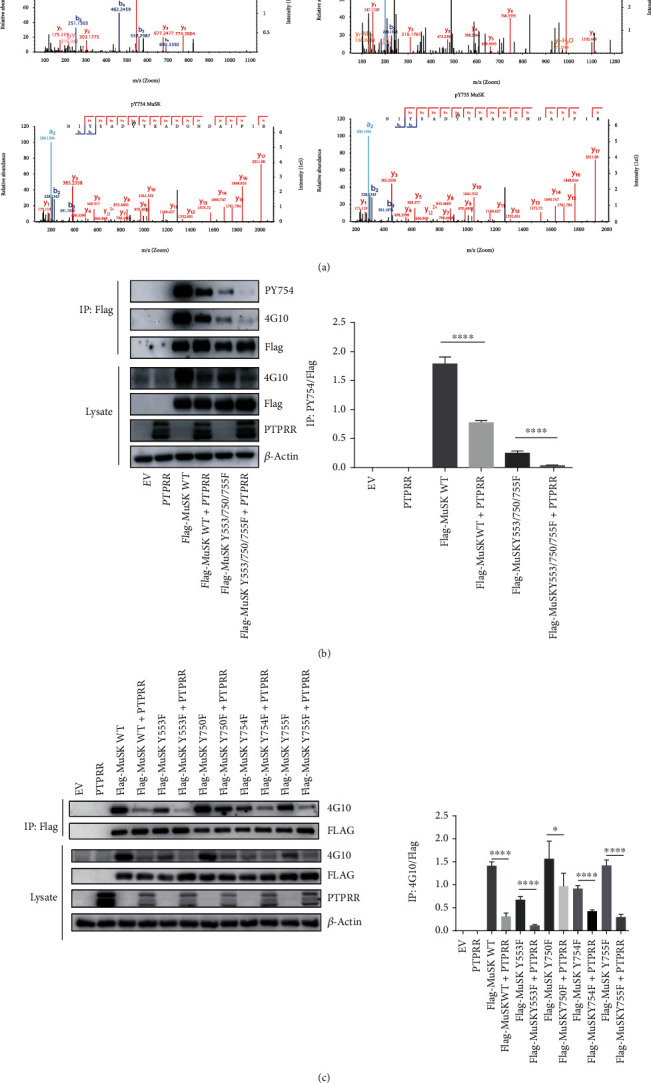
PTPRR dephosphorylated MuSK-Tyr754. (a) HEK293T cells were transiently transfected with Flag-MuSK or empty vector plasmids. Proteins were separated by SDS-PAGE and stained with Coomassie Blue. Bands corresponding to MuSK were excised, digested with trypsin, and analyzed by LC-MS. The LC peaks corresponding to the peptide fragments containing Tyr-553, Tyr-750, Tyr-754, and Tyr-755 in MuSK fractions were further analyzed by MS/MS. (b) HEK293T cells were transiently transfected with PTPRR, MuSK, or MuSK Y553/750/754F. MuSK was isolated by immunoprecipitation with anti-Flag antibody, probed with PY754 antibody to reveal pY-MuSK Y754, and probed with 4G10 to reveal total pY-MuSK. Lysates were probed with antibodies against MuSK, pY-MuSK, and *β*-actin. (c) HEK293T cells were transiently transfected with PTPRR, MuSK, MuSK Y553F, MuSK Y750F, MuSK Y754F, or MuSK Y755F. MuSK was isolated by immunoprecipitation with anti-Flag antibody and probed with 4G10 to reveal pY-MuSK. Lysates were probed with antibodies against MuSK, PTPRR, pY-MuSK, and *β*-actin. The data were obtained from at least three experiments. Data were shown as mean ± S.D. NS: no significant difference. ^∗^*P* < 0.05 and ^∗∗∗∗^*P* < 0.0001.

To determine the phosphorylated sites of MuSK, Flag-MuSK was overexpressed in HEK293T cells and purified from cell lysates by immunoprecipitation with Flag-Beads. Purified Flag-MuSK was detected by mass spectrometry. We identified Tyr-553, Tyr-750, Tyr-754, and Tyr-755 as the primary sites of MuSK phosphorylation (Figure 4(a)).

We generated Tyr to Phe triple mutations for C-terminal residues 750/754/755, 553/754/755, 553/750/755, and 553/750/754 of MuSK, which were cotransfected into HEK293T cells with PTPRR for 24 h. No phosphorylation was detected by pTyr (4G10) blotting for MuSK Y750/754/755F, Y553/754/755F, or Y553/750/754F (data not shown). MuSK Y553/750/755F, for which phosphorylation depends on Tyr754, showed attenuated phosphorylation compared with MuSK-WT. Additionally, we found that phospho-Tyr754 was dephosphorylated by PTPRR (Figure 4(b)). Unexpectedly, PTPRR profoundly diminished the phosphorylation of MuSK Y754F (Figure 4(c)). These results demonstrated that PTPRR could dephosphorylate MuSK-Tyr754.

### 2.5. PTPRR Significantly Reduced MuSK-Dependent AChR Clustering in C2C12 Myotubes

**Figure 5:**
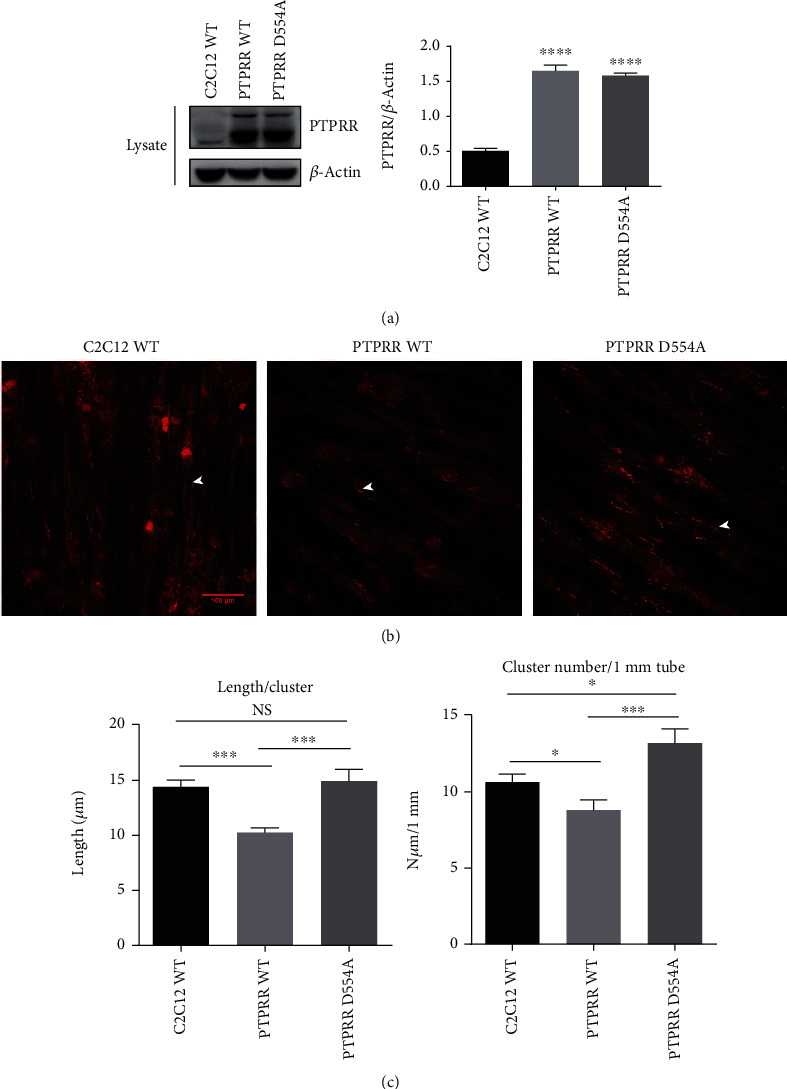
PTPRR significantly reduced MuSK-dependent AChR clustering in C2C12 myotubes. (a) C2C12 myoblasts were stably transfected with PTPRR, DA mutant, or empty vector plasmids. The efficiency of overexpression PTPRR was verified by western. (b) Compared with the C2C12 transfected with DA mutant or empty vector, C2C12 transfected with PTPRR had markedly reduced AChR clusters. White arrowheads indicate AChR clusters. (c) Quantification of AChR clusters ≥5 *μ*m in length from (b). The data were obtained from at least three experiments. Data were shown as mean ± S.D. NS: no significant difference. ^∗^*P* < 0.05, ^∗∗∗^*P* < 0.001, and ^∗∗∗∗^*P* < 0.0001.

PTPRR expression in C2C12 myotubes was highest after one day of differentiation (Supplementary Figure S2). To verify the hypothesis that PTPRR inhibits agrin-induced AChR clustering, monoclonal C2C12 cell lines overexpressing PTPRR-WT, PTPRR-D554A, or empty vector (LW009) were constructed. The efficiency of overexpression PTPRR was verified by western blot (Figure 5(a)). Compared with AChR clusters of WT or PTPRR-D554A C2C12, PTPRR-WT C2C12 was markedly reduced in both the number and length (Figures 5(b) and 5(c)). These results indicate that PTPRR significantly reduced MuSK-dependent AChR clustering in C2C12 myotubes.

## 3. Discussion

The formation and maintenance of NMJs ensures the efficient transmission of synaptic signals. Many neuromuscular diseases that present as neurological disorders, including MG and CMS, are due to deficits in NMJ formation or maintenance. The first key event in NMJ formation and maintenance is a high concentration of AChRs in the postsynaptic membrane. The redistribution of AChRs in muscle is modulated by a series of molecules including agrin, LRP4, MuSK, Src, tyrosine kinases, and phosphatases [13].

In NMJ formation, muscle fibers form primitive AChR clusters prior to the arrival of motor nerve terminals in process called muscle prepatterning [14]. New clusters are induced, and those in nonsynaptic areas disperse as the nerve terminals innervate muscle fibers. Tyrosine phosphorylation, which accumulates in muscle prepatterning, has been demonstrated to play an important role in the generative stages of AChR clusters [15–17]. AChR clustering can be inhibited by tyrosine kinase inhibitors such as RG50864. Labeled phosphotyrosine disappears before AChR clusters disassembly at the sites where AChR clusters disperse [13]. This suggests that tyrosine phosphatases function in restricting the spread of the activated kinase signal and thereby disperse AChR clusters. Zhao et al. [18] showed that tyrosine phosphatase Shp-2 regulates agrin-induced AChR clustering in vitro. Other studies have reported that NSC-87877, a Shp-2-specific inhibitor, increases MuSK phosphorylation and protects AChRs from the effects of MG MuSK antibody [19]. Nevertheless, NMJ formation and maintenance appeared normal in mice with Shp-2 conditional knockout in skeletal muscle [20]. These findings suggest that there are other tyrosine phosphatases that regulate NMJ formation and maintenance in muscle.

MuSK is essential for NMJ formation during embryogenesis and maintenance in adults [5]. Impairment of MuSK expression in adult mice leads to disassembly and destabilization of new synapses [21, 22]. Previous studies have shown that a MuSK mutation that impairs its kinase activity causes CMS [10]. Coincidentally, autoantibodies to MuSK are responsible for MuSK-dependent MG [11]. We found that conditional knockout of MuSK in C2C12 myotubes completely abolished the agrin-induced AChR clustering compared with wild-type myotubes. The intracellular region of MuSK contains four major tyrosine phosphorylation sites, three in the activation loop and one in the juxta-membrane region [23–26]. Here, we verified that MuSK tyrosine-553, tyrosine-750, tyrosine-754, and tyrosine-755 are the major tyrosine phosphorylation sites.

In this study, by combining the PTP substrate-trapping strategy with immunoprecipitation, we identified PTPRR as the tyrosine phosphatases responsible for dephosphorylating MuSK. PTPRR is a subfamily of classical PTPs and is considered as a tumor suppressor regulating proliferation or differentiation of cancer cells including oral squamous cell carcinoma, cervical cancer, breast tumor, and colorectal carcinomas [27–30]. We identified Tyr754 as the possible site of MuSK dephosphorylation by PTPRR through generating Tyr to Phe triple mutations for the C-terminal residues 553/750/755 of MuSK. Additionally, there are other candidate Tyr sites, considering that the Y754F mutation can still be dephosphorylated by PTPRR. However, these other candidate Tyr sites were hard to confirm by immunoblot because the triple mutations Y750/754/755F, Y553/754/755F, and Y553/750/754F did not show enough phosphorylation to detect. To confirm the candidate Tyr sites in future studies, we would perform MS analysis from C2C12 myotubes stably expressing PTPRR (active and inactive) to demonstrate MuSK dephosphorylation.

PTPRR-knockout mice display defects in motor coordination and balancing skills but display normal cerebellar morphological characteristics [31]. Using monoclonal C2C12 cell lines overexpressing PTPRR-WT or D554A, we found that PTPRR significantly reduced MuSK-dependent AChR clustering *in vitro*. The phenotype suggests that PTPRR-knockout mice may fail to form or maintain NMJs. However, it is a limitation for our study considering overexpressing PTPRR may cause artefacts or unphysiological response. Furthermore, Wang et al. [32] showed that PTPRR dephosphorylated and inactivated *β*-catenin as a tumor suppressor in ovarian cancer. Another study reported that *β*-catenin played a negative role in AChR clustering at NMJs [33]. That study suggests that PTPRR may also regulate AChR clustering by dephosphorylating *β*-catenin. To solve this problem, the construction of mouse model of skeletal muscle-specific PTPRR-knockout and related-MS analysis would be performed for further study. The formation and maintenance of NMJs would also be studied.

In summary, during NMJ formation and maintenance, tyrosine phosphatases function in restricting the spread of activated MuSK and thereby regulate AChR clustering. We determined that PTPRR is responsible for dephosphorylating MuSK-Tyr754. Moreover, we have shown that PTPRR significantly reduced MuSK-dependent AChR clustering in vitro. Consequently, inhibition of PTPRR may improve MuSK activity and offer an improved strategy for therapeutic intervention in MG and CMS.

## 4. Method and Materials

### 4.1. Reagents and Antibodies

PrimeSTAR® GXL DNA Polymerase, Premix Taq™ DNA Polymerase, and PrimeScript™ RT Master Mix were from TaKaRa. TransIT®-2020 Transfection Reagent was from Mirusbio. Fetal bovine serum was from Cellgro. Horse serum was from Gibco. Anti-phosphotyrosine antibody (4G10) was from Merck/Millipore. Anti-Flag-monoclonal antibody was from GNI. Anti-PTPRR antibody was from Thermo Fisher. Anti-MuSK phosphotyrosine (phospho Y754) antibody was from Abcam. Anti-MuSK antibody and agrin were from R&D. Alexa Fluor 555-conjugated *α*-BTX was from Promokine.

### 4.2. Plasmids and Transfection

Site-directed mutagenesis of MuSK and PTPRR was executed following the Agilent protocol. Generated mammalian expression plasmids were MuSK Y553F, Y750F, Y754F, Y755F, Y750/754/755F, Y553/754/755F, Y553/750/755F, Y553/750/754F (pFlag-cmv-MuSK), and PTPRR D554A (pcDNA3.1). Transient transfection followed the Mirus manufacturer protocol (Transit-2020, Mirus). Cells were harvested 24 h after transfection for further experiments.

### 4.3. Infection and FACS Sorting

The PTPRR-WT and D554A cDNA were subcloned into the LWT009-GFP vector using BamHI/NOTI restriction sites. Construction of PTPRR-overexpression or sgRNA knockout cell lines was via lentiviral infection. Specifically, lentivirus expressing PTPRR-WT (LWT009-GFP), D554A (LWT009-GFP), or CAS9-BFP was generated in 293T cells by cotransfecting the corresponding vector, PAX2, and VSVG at a ratio of 2 : 1 : 1. Forty-eight hours after transfection, viral medium was harvested and then incubated with C2C12. After reaching 30% confluence, GFP^+^ or BFP^+^ cells were subsequently purified by FACS sorting via the BD FACS Aria III flow cytometer. The sorted single clone C2C12 cell was cultured to expand the cell population. The overexpression of PTPRR-WT and D554A was confirmed by immunoblotting with anti-PTPRR antibody.

MuSK^−^ single clone C2C12 was generated by the CRISPR/Cas9 system. We used the online software (CRISPR gRNA Design tool: https://www.atum.bio/eCommerce/cas9) to design the sgRNA for targeting MuSK genes. The sequences were as follows: sgRNA1, 5′-AAGTTTCTCAGTCCCGCTG-3′, and sgRNA2, 5′-AGCATTGTCCCCCTTGATCC-3′. The complementary oligos of the sgRNAs were synthesized, phosphorylated, and incubated at 37°C for 1 h. The pairs of complementary oligos were mixed and PCR was performed: 95°C for 5 min, −10°/cycle, 0.1°/second, back to step one X7, 25°C 20 min, and 4°C hold. The MP783-GFP plasmid was digested with AarI and then ligated with the PCR product. The resulting MP783-GFP-sgRNA plasmid for targeting MuSK was confirmed by sequencing. CAS9-BFP C2C12 was infected with viral packaging MP783-GFP-sgRNA plasmid as previously mentioned. The CAS9-BFP/MP783-GFP-sgRNA double positive stable C2C12 was sorted by flow cytometry. The sorted single clone cells were expanded in culture, and the efficiency of MuSK knockout was validated by immunoblotting.

### 4.4. Immunoblotting and Immunoprecipitation

Cells were lysed with lysis buffer (20 mM Hepes pH 7.5, 150 mM NaCl, 1% NP40, 50 mM NaF, 1 mM Na_3_VO_4_, 10% glycerol, protease inhibitor cocktail from Roche) at 4°C for 30 min. After the concentration was determined, proteins were separated by SDS-PAGE and transferred to nitrocellulose membranes. Membranes were blocked and then incubated for 2 h at room temperature with primary antibody. Proteins were detected with horseradish peroxidase-conjugated secondary antibodies (Genscript) and ECL (Pierce).

For immunoprecipitation, precleared cell extracts were incubated with the indicated antibody for 4 h in a cold room with rotation followed by 1 h of pulldown by 1 : 1 Protein A/G-agarose beads. Immunoprecipitate was washed three times with lysis buffer on a rotating wheel at 4°C for 5 min before SDS-PAGE and immunoblotting.

### 4.5. Mass Spectrometry

Flag-MuSK was overexpressed in HEK293T cells and purified from cell lysates by immunoprecipitation with Flag-Beads. Purified Flag-MuSK proteins were then analyzed by mass spectrometry on a Thermo Fisher Scientific LTQ XL ion trap mass spectrometer.

### 4.6. C2C12 Myotube Culture and AChR Cluster Analysis

C2C12 myoblasts were propagated in Dulbecco's modified Eagle medium (DMEM) containing 4.5 g/l D-glucose and pyruvate, supplemented with 20% fetal bovine serum and 1% penicillin/streptomycin (growth medium). After reaching 90% confluence, differentiation was then induced by switching the growth medium to differentiation medium containing DMEM, 2% horse serum, and 1% penicillin/streptomycin. The differentiation medium was refreshed daily. Contracting myotubes were usually observed after 3 days and then used for further experiments. Cells were maintained at 37°C in an atmosphere of 5% CO_2_.

AChR clusters of C2C12 myotubes were induced by application of agrin for 24 h. To visualize AChR clusters, C2C12 myotubes were fixed in 4% paraformaldehyde for 15 min, stained with Alexa Fluor 555-conjugated *α*-BTX, rinsed with phosphate buffered saline, and viewed under a confocal microscope (Zeiss LSM 710 NLO). AChR clusters were analyzed for number and length (≥5 *μ*m) using ImageJ software.

### 4.7. Statistical Analysis

Statistical tests were performed with GraphPad Prism 6 as indicated in the figure legends. Error bars represent the standard error of the mean unless otherwise stated. Unpaired Student's *t*-test was used to compare data between two groups. Differences were considered significant at *P* < 0.05.

## Data Availability

The data used to support the findings of this study are included in the article/Supplementary Material. Further inquiries can be directed to the corresponding authors.

## Supplementary Materials

Supplementary Materials Supplementary Figure S1: HEK293 T cells were cotransfected with flag-musk and various 10 tyrosine phosphatases as indicated. Tyrosine phosphorylation of MuSK, actin, and various PTPS was probed. Supplementary Figure S2: C2C12 cells were induced to differentiate for various lengths of time without and with agrin-stimulative for one day (as indicated). PTPRR expression was highest after one day of differentiation.
