# Supplementary material for: Protein Tyrosine Phosphatase Receptor Type R (PTPRR) Reduces AChR Clustering by Dephosphorylating MuSK
Source: Dis Markers. 2022 Sep 5;2022:5160624. doi: 10.1155/2022/5160624 (PMC9467777; doi:10.1155/2022/5160624)
Supplement: Supplementary Materials — Supplementary Figure S1: HEK293 T cells were cotransfected with flag-musk and various 10 tyrosine phosphatases as indicated. Tyrosine phosphorylation of MuSK, actin, and various PTPS was probed. Supplementary Figure S2: C2C12 cells were induced to differentiate for various lengths of time without and with agrin-stimulative for one day (as indicated). PTPRR expression was highest after one day of differentiation. [file 5160624.f1.docx]

Supplementary Material

## Supplementary Figures


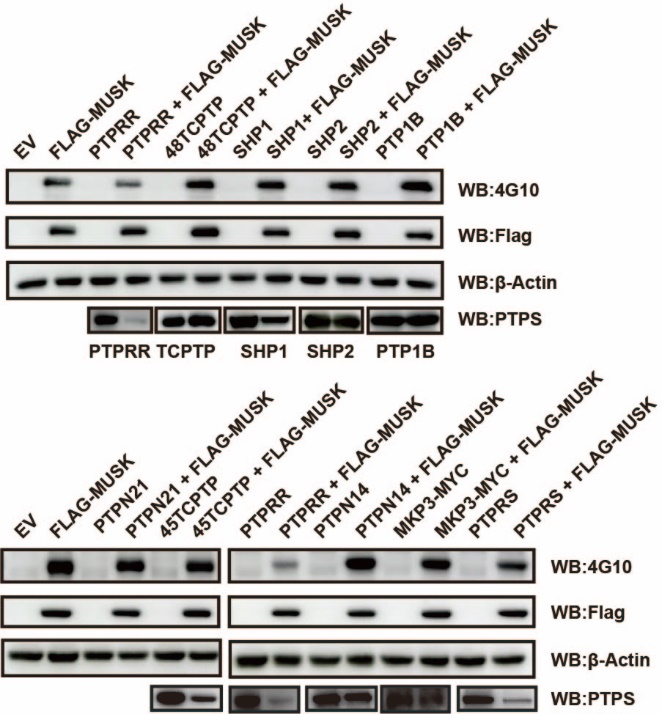


**Supplementary Figure S1.** HEK293 T cells were co-transfected with flag-musk and various 10 tyrosine phosphatases as indicated. Tyrosine phosphorylation of MuSK , MuSK, actin and various PTPS were probed.


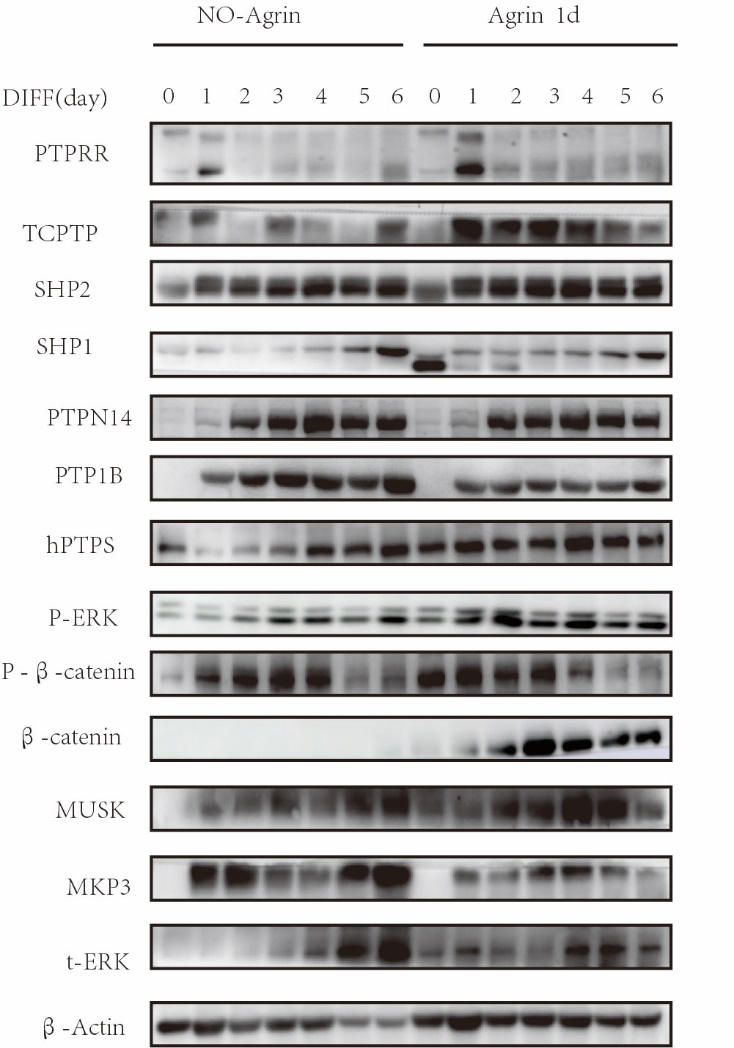


**Supplementary Figure S2.** C2C12 cells were induced to differentiate for various lengths of time without and with agrin-stimulative for one day (as indicated). PTPRR expression was highest after one day of differentiation.

、
